# The impact of post-traumatic stress on the mental state of university hospital physicians – a cross sectional study

**DOI:** 10.1186/s12888-022-03719-3

**Published:** 2022-02-03

**Authors:** Christian Bock, Tanja Zimmermann, Kai G. Kahl

**Affiliations:** 1 Department of Occupational Safety, Hannover Medical School, Hannover, Germany; 2 Department of Psychosomatic Medicine and Psychotherapy, Hannover Medical School, Hannover, Germany; 3 Department of Psychiatry, Social Psychiatry and Psychotherapy, Hannover Medical School, Hannover, Germany

**Keywords:** Post-traumatic stress_1_, Physicians_2_, Risk assessment_3_, Depression_4_, Anxiety_5_, ptsd_6_

## Abstract

**Background:**

Hospital physicians have an increased risk for post-traumatic stress caused by work-related trauma. This study examines the frequency of reported traumatic events (TE), post-traumatic stress (PTS) and its possible consequences for the mental state and work ability of physicians at a university hospital.

**Methods:**

As part of the mandatory psychological risk assessment, *n* = 145 physicians (*n* = 56 female; 38.6%) were examined at a university hospital in Germany in a cross sectional study. TE, PTS and symptoms of depression and anxiety were assessed using the self-report questionnaires “Freiburger Screening Fragebogen to identify patients at risk for the development of a post-traumatic stress disorder in the group of severely injured patients” (PTBS-13), the “Patient Health Questionnaire” (PHQ-2) and the “Generalized Anxiety Disorder scale” (GAD-2). Work ability was assessed using a modified version of the questionnaire for workplace analysis (KFZA). The response rate was 52%.

**Results:**

Traumatic events were experienced by *n* = 125 physicians (86.2%) throughout their whole career. Of these, 19 physicians (15.2%) reported PTS. PTS is reported by 12 of 56 female physicians (63.2%), compared to 7 of 89 reports of PTS by male physicians (36.8%). Physicians with PTS symptoms had higher depression scores (*p* = 0.007) compared to physicians without TE or with TE, but without PTS. Physicians with PTS reported significantly reduced work ability caused by constantly interrupted work (*p* = 0.03). Female gender was the greatest risk factor for the development of PTS. (*p* = 0.001).

**Conclusions:**

Physicians – especially females – with PTS may have an increased risk of developing depressive symptoms. Therefore, interventions aimed at reducing trauma-related stress symptoms may be helpful in improving mental health of hospital physicians. Further studies with more physicians from different hospitals are necessary to support the results.

## Introduction

The implementation of psychological risk assessment has been a legal obligation for all German companies since 2013 [4]. A psychological risk assessment can include job strain factors like workload, time pressure, work interruptions and conflicts as well as the exposure to traumatic events. The improvement of mental health at work is ethically important and a relevant competitive and economic factor as well [26]. High job strain can lead to significant sickness leave [42]. Several studies [27, 29, 33, 34, 39] describe the effects of job strain and mental health in the general population. Regarding physicians, negative effects of job strain on mental health can be depressive symptoms as described by [30] and Ruotsalainen et al. 2014 [28]. In general, hospital physicians have to deal with highly demanding working conditions as the experience of emotionally demanding situations. In addition to the impact of job strain on mental health, several studies show that the exposure to work-related critical incidents such as moribund patients or violence may cause symptoms of traumatic stress or a post-traumatic stress disorder (PTSD) [6, 15, 44]. According to Van Eerd et al. [41], many occupations, including those of health care professionals, lead to exposures which could be associated with PTSD. Skogstad et al. [35] estimate the prevalence for lifetime PTSD among firefighters and ambulance personnel (up to 20%), health care professionals (“high rates of PTSD symptoms”) and police officers (“less than 10%”). Other professions subjected to work-related traumatic events include train drivers, divers, journalists, sailors and employees in bank, post offices or in stores (ibid.). Key symptoms for PTSD include intrusion/re-experiencing of trauma, avoidance, negative cognitions as well as mood and hyper-arousal [16]. Additionaly, stress “resulting from helping or wanting to help a traumatized or suffering person” is defined as secondary traumatic stress (STS) ([9], p. 7).

Lo et al. [20] describe higher rates of PTSD among resident physicians compared to the rates of PTSD in the general population in the USA and Canada. Scheepstra et al. [31] describe a higher point prevalence rates for PTS among Dutch physicians after traumatic events and higher rates for depression and anxiety symptoms especially among female physicians, compared to the 12-months prevalence of depression and anxiety among the general population in the Netherlands. Axisa et al. [3] describe a high risk for depression, stress and anxiety as well as exceptionally high secondary traumatic stress (STS) among Australian physician trainees. Lazarus (2014) [19] presumes that the cumulative stress of practice, including the exposition to primary and secondary trauma, may cause PTSD. Among German emergency physicians and paramedics, Eiche et al. [8] found high rates of screened possible depression indicated by a WHO-5 well-being score below 50% (43.4% of the participants) and a positive PTSD screening result of 5.4%, associated with significantly lower well-being. The association between PTSD and a low WHO-5 well-being score (< 28%) was significant (ibid.). In the group of German emergency medical physicians, Pajonk et al. [24] report that 16.8% of the participants meet the criteria for probable PTSD, 4.1% for burnout, and 3.1% for clinical depression, but see a better indicator for PTSD in the physicians’ personality than in the exposition to traumatic events. Finally, Greinacher et al. [10] found low levels of secondary traumatization in first responders.

In a systematic review among psychiatric nurses and other healthcare workers, Hilton et al. [12] describe associations between PTSD and burnout, poor mental health, neuroticism, low compassion satisfaction and compassion fatigue. Most studies showed no significant association between gender and PTSD, so the relation between these factors is reported as “suggestive” (ibid.). Age and years of working experience are described as mostly not to be associated with PTSD symptoms. (ibid.). Additionally, more than workplace related reasons may cause PTS and PTSD, such as accident experiences, (sexual) abuse, physical attacks or unexpected deaths of relatives or friends [14].

Previous studies refer to higher risks for depression, anxiety and PTSD for physicians, but effects of traumatic events and post-traumatic stress on the mental state of hospital physicians, especially regarding symptoms of depression and anxiety, have rarely been examined so far. This cross-sectional study examines the association between traumatic events, post-traumatic stress (PTS) and symptoms of depression and anxiety (as well as possible influences of demographic factors) among physicians in a university hospital. The survey was part of a mandatory psychological risk assessment. The key focus is on PTS resulting in flashbacks as one of the key symptoms of PTSD, not on the full expression of PTSD, following traumatic events (TE). The main hypothesis is that hospital physicians suffering from PTS have a higher risk of developing symptoms of depression and anxiety. Confirmation of this hypothesis could lead to an improvement in the hospitals’ services offered to clinical staff to support their mental health, reduce sickness leave and improve work ability. Previous findings of Bock et al. [5] indicate an influence of STS on nurses’ mental health and higher job strain of nurses with STS. Hence, further hypotheses are that the physicians’ experience of TE/PTS has an influence on different job strain factors as well. Furthermore we explored if associations between TE/PTS and the physicians’ age, gender and medical specialty can be found.

## Methods

### Participants

The cross-sectional study was approved by the ethics committee of Hannover Medical School (no. 2975–2015) and staff committee of the hospital. Psychological risk assessment was performed in *n* = 1057 employees working in different professions and departments at Hanover Medical School by an occupational psychologist of the Department of Occupational Safety. The physicians’ response rate was 52%, the overall response rate 65%.

### Procedure

The participants were asked to fill in an anonymous questionnaire online within three weeks as a part of the psychological risk assessment process. All participants were informed about the study content and only filled in the questionnaire if they agreed to participate in the study. They were grouped by profession and department. The departments included were selected by random. Of the 1057 participants, *n* = 145 were physicians with *n* = 56 females (38.6%), working in different disciplines at the Hanover Medical School, such as internal medicine, surgery, paediatrics, psychiatry, psychotherapy and psychosomatic medicine. The objective of the study was to focus on physicians only, therefore other professions, such as nurses, administration staff, etc. were excluded from this study. Eligibility criteria for participating in this study were not defined.

### Measures

Post-traumatic stress (PTS) was assessed using two items of the “Freiburger Screening Fragebogen to identify patients at risk for the development of a post-traumatic stress disorder in the group of severely injured patients” (PTBS-13, [32]) with an internal consistency of α = .82. Participants were asked whether they had experienced traumatic events at work (yes/no) and whether they currently suffered from flashbacks regarding traumatic events (STE) at work (yes/no). These data were used to classify subjects based on TE/PTS into three distinct groups. If no traumatic work event was reported, subjects were classified as the “no TE” group (*n* = 16). If a traumatic event at work was reported without flashbacks, subjects were classified as traumatic experience without post-traumatic stress, the “TE without PTS” group (*n* = 106). If flashbacks were affirmed in the context of this traumatic experience, subjects were classified into the “TE with PTS” group (*n* = 19).

Work ability was assessed using a modified version of the “Questionnaire for workplace analysis” (KFZA) by Prumper et al. [25] with a scale ranging from 1 to 5. The included scales were workload (two items, α = .70), scope of action (two items, α = .70), social support (two items, α = .72), feedback (two items, α = .64), work environment (two items, α = .60), information and participation (two items, α = .70) and emotional requirements (two items, α = .63). Participants were asked if they “strongly agree”, “agree”, “neither agree nor disagree”, “disagree” or “strongly disagree” to statements such as “I can count on my colleagues if difficulties at work should arise”.

Symptoms of depression and anxiety were assessed using the two item Patient Health Questionnaire-2 (PHQ-2 [21];) and the two item Generalized Anxiety Disorder scale (GAD-2 [36];). Both scales pertain to symptom frequency during the last two weeks using a 4-point Likert scale (0–3) ranging from “Not at all” (0) to “Almost every day” [3]. Scores for both questionnaire range from 0 to 6, with ≥3 being used as the optimal cut-off point for screening purposes. The PHQ-2 assessed the frequency of depressed mood or anhedonia. Sum scores ≥3 suggest a major depressive disorder. The internal consistency in the current sample was α = .72. According to Arroll et al. [1], the PHQ-2 was validated in primary care patients. The authors point out that “sensitivity and specificity of the PHQ-2 for diagnosing major depression were 86% and 78%, respectively, with a score of 2 or higher and 61% and 92% with a score 3 or higher” (ibid.).

The GAD-2 assessed experienced feelings of nervousness, anxiety or uncontrollable worry. Sum scores ≥3 suggest the presence of an anxiety disorder. The internal consistency in the current sample was α = .78. The GAD-2 was validated in primary care patients and, according to Kroenke et al. [18] “performed well (area under the curve, 0.80 to 0.91) as screening tools for all 4 anxiety disorders”.

Age was measured on a 5-point ordinal scale consisting of the response options “up to 25 years”, “26–35 years”, “36–45 years”, “46–55 years” or “56 years or more”.

The marital status was measured on a 4-point nominal scale with the response options “single”, “married/partnership”, “divorced” or “widowed”. In order to measure differences in the experience of traumatic events and post-traumatic stress between physicians of different departments, physicians were grouped into the specialties “internal medicine”, “surgery”, “paediatrics” and “psychiatry/psychosomatics”.

### Data analysis

All statistical analyses were conducted using SPSS version 25. Normal distribution was checked by conducting a Shapiro-Wilk-Test and a Kolmogorov-Smirnov-Test. As a result, the sample was not distributed normally. Therefore a nonparametric Kruskal-Wallis Test with a Bonferroni correction as post-hoc-test was conducted to measure differences in the central tendency of the dependent and independent variables. Work ability factors, depression risk and anxiety risk were set as dependent variables and TE/PTS was set as independent variable with the three levels “no TE”, “TE without PTS” and “TE with PTS”.

Descriptive analyses included gender, age, marital status and department affiliation. Group differences concerning nominal variables were compared using Chi square tests. Group differences between “gender” as independent variable and symptoms of depression and anxiety as dependent variables were measured using an independent sample t-test. A one-way between subjects ANOVA was conducted to compare the effects of age on symptoms of depression and anxiety.

All tests were based on a significance level of *p* = 0.05.

## Results

### Sample

Fifty-nine percent of the sample was male and most (46.2%) were in the age range between 26 and 35 years. Seventy-six (52.4%) were working in internal medicine, twenty-nine (20.0%) in pediatric medicine, twenty-one (14.5%) in psychiatric medicine and nineteen (13.1%) in surgery. Most physicians (79.3%) were in a relationship (Table 1).

**Table 1:** Psychosocial and occupational data (*N* = 145)

Factor	All physicians (N/%)
Gender^a^	
Female	56 (38.6%)
Male	86 (59.3%)
Age range^b^	
≤25	1 (0.7%)
26–35	67 (46.2%)
36–45	48 (33.1%)
46–55	19 (13.1%)
≥56	6 (4.1%)
Physician Specialty	
Surgery	19 (13.1%)
Internal Medicine	76 (52.4%)
Pediatrics	29 (20.0%)
Psychiatry	21 (14.5%)
Traumatic event (TE)	125 (86.2%)
Post-traumatic stress (PTS)	19 (15.2%)
Marital status^c^	
Single	21 (14.5%)
Married/partnership	115 (79.3%)
Divorced	6 (4.1%)
Widowed	0

Missing values: ^a^3 (2.1%); ^b^4 (2.8%); ^c^3 (2.1%)

### Traumatic events and its influence on depression and anxiety

Traumatic events (TE) were reported by 128 physicians (88.3%), of whom 19 (13.1%) reported TE followed by recurring memories and/or flashbacks (Table 1). Female physicians (*n* = 12; 21.4%) reported PTS more often than male physicians (*n* = 7; 8.2%); (χ^2^ (2,141) = 13.3, *p* = 0.001). 78% of all physicians under 46 years of age experience more traumatic events than physicians equal to or over 46 years (64% of all physicians ≥46 years) (χ^2^ (8,140) = 16.8, *p* = 0.03). There was no significant association between the physicians’ department affiliation and TE/PTS (χ^2^ (6,144) = 7.3, *p* = 0.29) (Table 2).

**Table 2:** Descriptive statistics for gender, age, physician specialty, PHQ-2, GAD-2, work strain and work ability dependent on traumatic experiences (no TE, TE without PTS, TE with PTS^1^)

	No TE (N = 16)	TE without PTS (*N* = 106)	TE with PTS (*N* = 19)
Gender (n, %)			
Female	11 (19.6%)	33 (58.9%)	12 (21.4%)
Male	5 (5.9%)	73 (85.9%)	7 (8.2%)
Age range (n, %)			
≤25	1 (100%)	0	0
26–35	6 (9.1%)	53 (80.3%)	7 (10.6%)
36–45	4 (8.3%)	37 (77.1%)	7 (14.6%)
46–55	4 (21.1%)	10 (52.6%)	5 (26.3%)
≥56	0	6 (100%)	0
Physician Specialty (n, %)			
Surgery	3 (15.8%)	13 (68.4%)	3 (15.8%)
Internal Medicine	6 (8.0%)	61 (81.3%)	8 (10.7%)
Pediatrics	3 (10.3%)	19 (65.5%)	7 (24.1%)
Psychiatry	4 (19.0%)	16 (76.2%)	1 (4.8%)
PHQ-2 (M, SD)	1.1 ± 1.5	1.2 ± 1.2	2.3 ± 1.7 ^ab^
GAD-2 (M, SD)	1.3 ± 1.7	1.3 ± 1.2	2.2 ± 1.9
Workload (M, SD^2^)			
Being under pressure	4.0 ± 0.8	4.0 ± 0.9	4.1 ± 0.9
Having too much work	3.8 ± 1.0	3.8 ± 0.9	3.9 ± 1.2
Control over work (M, SD)			
Influence on amount of work	3.6 ± 1.3	3.6 ± 1.1	3.6 ± 1.3
Plan work independently	3.3 ± 1.2	3.1 ± 1.1	3.5 ± 1.2
Social support (M, SD)			
Support by colleagues	2.3 ± 1.3	2.2 ± 0.9	2.3 ± 1.2
Support by supervisors	2.9 ± 1.3	2.6 ± 1.1	2.8 ± 1.3
Workflow (M, SD)			
Information or equipment not available	2.7 ± 1.1	2.5 ± 0.9	2.5 ± 1.1
Work often interrupted	3.8 ± 1.1	4.2 ± 0.9	4.6 ± 0.8 ^ab^
Feedback (M, SD)			
Appropriate feedback by colleagues	2.9 ± 0.9	3.1 ± 1.0	3.0 ± 1.3
Appropriate feedback by supervisors	3.6 ± 1.1	3.5 ± 1.1	3.5 ± 1.1
Work environment (M, SD)			
Stressful work environment	2.4 ± 1.0	3.1 ± 1.3	3.1 ± 1.3
Equipment inadequate	2.9 ± 1.5	3.4 ± 1.3	3.2 ± 1.6
Information and participation (M, SD)			
Always kept up-to-date	2.9 ± 1.2	2.8 ± 1.0	3.1 ± 1.1
Superiors consider employees ideas	2.8 ± 1.4	3.0 ± 1.1	3.0 ± 0.9

^a^*Means a group difference (p < 0.05) between “TE with PTS” and “no TE”.* ^b^*Means a group difference (P < 0.05) between “TE with PTS” and “TE without PTS”*

^*1*^*TE = traumatic events; PTS = post-traumatic stress*

*M mean, SD standard deviation*

**Fig. 1:**
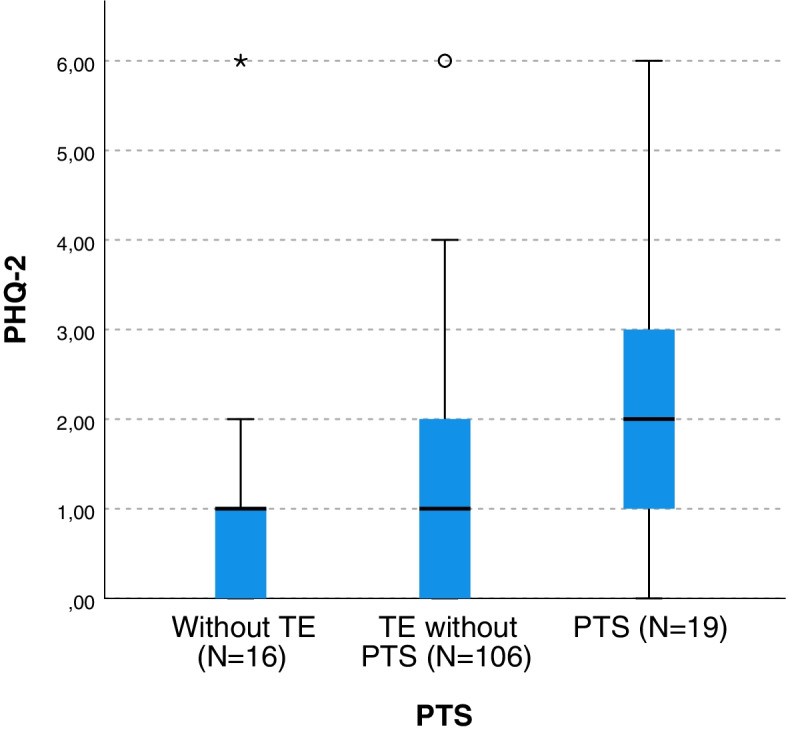
Increased Symptoms of depression in physicians who reported post-traumatic stress (PTS) compared to both other groups (no TE and TE/traumatic events without PTS). * Indicates a significance level *p* = 0.007

Physicians with PTS had higher depression scores (MR = 97.03) compared to physicians with no TE (MR = 61.03) and to physicians with TE without PTS (MR = 67.78; H (2,141) = 9.994, *p* = 0.007). In a pairwise comparison, the “no TE” group was significantly different from then “TE with PTS” group (*p* = 0.008) and the “TE without PTS” group was significantly different from the “TE with PTS” group (*p* = 0.003). The “no TE” group and the “TE without PTS” group did not differ significantly (*p* = 0.53) (Fig. 1).

Regarding the effect of PTS on the physicians’ anxiety risk, no significant differences occurred between physician with TS, no TE or with TE without PTS (H (2,141) = 5.129, *p* = 0.077).

### Post-traumatic stress and work factors

Physicians in the “TE with PTS” group (MR = 88.11) compared to physicians in the “TE without PTS” group (MR = 69.66) and to physicians with no TE (MR = 55.09) showed higher scores for “Work is often interrupted” (H (2,141) = 6939, *p* = 0.031).

Physicians with no TE, with TE without PTS and with TE and PTS showed no significant difference in the job strain factors scores of “workload” (H (2,143) = 0.42, *p* = 0.81), “scope of action” (H (2,144) = 1.53, *p* = 0.47), “social support” (H (2,143) = 0.12, *p* = 0.94), “feedback” (H (2,143) = 0.9, *p* = 0.96), “work environment” (H (2,142) = 2.86, *p* = 0.24) and “information and participation” (H (2,141) = 0.45, p = 0.8).

### Depression, anxiety and demographic factors

There was a statistically significant difference between anxiety among female physicians (M = 1.75, SD = 1.76) compared to male physicians (M = 1.16, SD = 1.07), *t*(136) = 2.44, *p* = 0.016. Apart from that, no other associations between depression, anxiety and demographic factors were significant.

## Discussion

Psychological risk assessment has been mandatory for all companies and professions in Germany since 2013, although only few companies have fully implemented it so far. We can only speculate that it has not been commonly accepted in hospitals so far to ask about physicians’ mental health. We expect different results compared to local physicians because of a different quality and quantity of job strain in hospitals and the higher exposure to potential traumatic events. Further studies could describe such a comparison.

This study describes the association between traumatic experiences (TE), post-traumatic stress (PTS) and symptoms of depression and anxiety, as well as their relation to work strain factors and demographic factors among university hospital physicians. High rates (> 88%) of self-reported traumatic experiences in physicians of different specialties occurred. Physicians with PTS (> 13%) described more symptoms of depression and had higher work strain rates regarding “Work often interrupted”. PTS is reported by 12 of 56 female physicians (63.2%), compared to 7 of 89 reports of PTS by male physicians (36.8%), so female physicians report PTS more frequently. Physicians under 46 years of age experience more trauma than physicians equal to or over 46 years.

The results indicate that physicians with PTS show significantly more symptoms of depression than physicians with no TE or physicians with TE without PTS.

Our results are in line with Eiche et al. [8] who report a significant association between PTSD and possible depression among paramedics and emergency physicians. Although the surveys used and the medical professions are not fully comparable, both results indicate that mental health and PTSD are connected with each other. These results are supported by a described association between PTSD and poor mental health [12]. Furthermore, our results expand the study by Scheepstra et al. [31] who found higher PTSD-, depression- and anxiety scores among Dutch physicians, but did not measure potential connections between PTSD and mental health. Scheepstra et al. describe the depression- and anxiety scores as significantly higher for females, which is partly in line with our result of significantly higher anxiety scores among female physicians. Furthermore, the absence of peer support is outlined as significantly associated with a more probable PTSD (ibid.). In line with Auxéméry [2] who describe additional risk factors for developing PTSD, such as younger age at the time of the trauma and pre-existing anxiety or depressive disorders, it is not possible to make a general statement if physicians with PTS have a higher risk for depression symptoms or if pre-existing anxiety or depressive disorders increase the risk for PTS after traumatic experiences at work. These findings have to be specified in further representative studies including more physicians. Additionally, future studies should assess other potential factors for PTS such as pre-existing mental disorders.

Furthermore, the influence of secondary traumatic stress (STS) on the mental state of hospital physicians has to be discussed. Bock et al. [5] describe higher depression scores among University Hospital nurses with STS. In line with Hamama et al. [11], who found no difference between nurses’ and physicians’ STS, it is questionable, if the results of this study show indications for STS among hospital physicians with an influence on their mental state as well. Further studies with more precise questions measuring STS could lead to more precise results.

The reasons for developing depression symptoms are multidimensional. An exposition to TE can be only one of several risk factors, including the individual mental constitution and working environment of each physician. Rau & Henkel [27] describe several work strain factors related to an increasing depression and anxiety risk. A noticeable result of our study is the higher score for the job strain factor “Work is often interrupted” of physicians in the “TE with PTS” group compared to the other groups (TE without PTS and no TE). Could this specific job strain factor influence a decrease of resilience, causing a higher vulnerability for PTS after a TE? This hypothesis could be examined in future studies.

Low social support is, according to Ruotsalainen et al. [28], one of the main risk factors among physicians for developing depression. Takizawa et al. [17, 30, 37, 40] refer to the supportive effect of social support on mental health. Scheepstra et al. [31] describe the absence of peer support after work-related traumatic events as a main risk for physicians’ developing a PTSD. A moderating effect of social support on the risk of developing symptoms of depression among physicians with PTS is plausible, but could not be proven in this study due to a limited number of physicians taking part. Therefore we did not calculate a moderation variable “social support” as part of a regression analysis, but we consider further studies to explore the effect of “social support” as a potential moderating variable.

Female physicians reported PTS more often than male physicians. These findings are in line with Auxéméry [2] and Scheepstra et al. [31] who describe higher risks of developing PTS for persons of female gender. Following our findings that physicians with PTS show depression symptoms, the female physicians’ depression risk is higher than the risk for male physicians. This assumption is supported by general findings of higher depression risks for females than for males [13]. However, gender is not a consistent risk factor for PTSD, as other studies describe higher risks for males [43] or no significant differences in gender [5, 22, 38]. Although we found a significant association between anxiety and gender, it was not possible to use “gender” as covariate because of a violation of statistical requirements. Future studies could lead to more precise findings of associations between gender and PTS/PTSD.

According to the current results, there was a significant association between the physicians’ age and TE/PTS. Younger physicians acting as residents are presumably more often the first physicians in emergencies, compared to senior physicians. Therefore, they might be exposed to traumatic events more often than older physicians. But it is questionable, if age really is the decisive factor or if work experience and career level are the main influences on TE/PTS. Dyrbye, Varkey et al. [7] point out the importance of regarding job related stressors in relation to different career levels of physicians. The current study did not differentiate between the physicians’ different career levels. It might be useful to put specific risk factors for different ages and career stages in concrete terms in further studies.

### Implications for practice

Based on the current findings, several comprehensive offers for physicians of all career levels are recommended in order to receive continuous mental support. All hospitals are recommended to offer possibilities of crisis intervention to physicians after a traumatic work-related event, especially younger and female physicians, such as” Critical Incident Stress Management “(CISM) programs. According to Müller-Leonhardt, Mitchell, Vogt & Schürmann [23], p. 172) “findings demonstrate that the adaptation of the CISM program in general hospitals takes time but, once established, it may serve as a mechanism for changing professional culture, thereby permitting the framing of even small incidents or near misses as an opportunity to provide valuable feedback to the system”. In general, structures of social support among colleagues are suitable to support resilience and mental health. Hamama et al. [11] suggest supportive senior–junior groups and the development of in-service training workshops for supportive communication behaviours especially towards professional identity formation among physicians. Work-related traumatization should not be regarded as a lack of resilience in physicians, but as an occupational hazard of the physicians’ work situation (cf. [11]). The issuing of measures to reduce traumatic stress symptoms is a chance for hospitals to promote mental health, work ability and commitment to the company by their employed physicians. Further programs to reduce job-related stress, especially constant work interruptions, could be an additional improvement for the physicians’ mental health. Moreover, longitudinal studies could identify long term effects of TE on PTS.

Finally, at the political level it could be discussed, if a mandatory psychological risk assessment leads to an improvement of the employees’ mental health in other countries as well.

### Limitations

The study has certain limitations. Subjective interpretation of the traumatic stressor and their nature were not assessed. Furthermore, due to the limited number of questioned physicians, and the limited number of medical specialties, the survey is non-representative for physicians in general. Consequently, due to the physicians’ unequal distribution in the experience of TE and PTS and the cross-sectional design of the study, only associations can be reported. Validated, but brief scales are only suitable to report tendencies, which must be validated in further studies. Regarding the response rate of 52% and despite the possibility to report anonymously, a substantial response bias due to socially desirable responding is likely. The presented data was taken from a psychological risk assessment, which is a legal obligation for German companies. The questionnaire used here had to be approved by the Employee Committee. Some potentially interesting facets of the results, such as personality traits, coping mechanisms and ability for the job, were not approved. Further studies including more physicians are necessary to validate the current findings.

## Conclusions

The current study found a difference between the exposure to work-related traumatic situations (“traumatic event/TE”) and flashbacks following work-related traumatic situations (“post-traumatic stress/PTS”) in association with depression symptoms. The physicians’ risks of experiencing symptoms of depression were significantly higher if flashbacks were affirmed. The results indicate the need for physicians’ workplace support such as crisis intervention and good structures of social support among colleges.

Due to a limited study population, further studies are needed, including more physicians from different faculties, settings and career levels, to draw firm conclusions.

## Data Availability

The data that support the findings of this study are available from the corresponding author upon reasonable request.

## Abbreviations

PTS: Post-traumatic stress
PTSD: Post-traumatic stress disorder
STS: Secondary traumatic stress
TE: Traumatic event
